# Efficacy of Safinamide and Gender Differences During Routine Clinical Practice

**DOI:** 10.3389/fneur.2021.756304

**Published:** 2021-12-14

**Authors:** Maria T. Pellecchia, Marina Picillo, Maria C. Russillo, Maria F. De Pandis, Erminio Bonizzoni, Ivan Marjanovic, Carlo Cattaneo

**Affiliations:** ^1^Department of Medicine, Surgery and Dentistry “Scuola Medica Salernitana”, Neuroscience Section, University of Salerno, Fisciano, Italy; ^2^Clinical Trial Center Parkinson, San Raffaele Cassino, Cassino, Italy; ^3^Section of Medical Statistics and Biometry “GA Maccacaro”, Department of Clinical Science and Community, University of Milan, Milan, Italy; ^4^Medical Department, Zambon SpA, Bresso, Italy

**Keywords:** Parkinson's disease, motor fluctuations, safinamide, gender differences, real-life evaluation

## Abstract

**Background:** There is increasing evidence of gender differences in the epidemiology and clinical manifestation of both motor and non-motor symptoms of Parkinson's disease (PD). Nevertheless, few data are available on gender differences in the response to antiparkinsonian drugs. Safinamide is a multimodal drug with positive effects on motor and non-motor fluctuations that might improve patients' care and quality of life.

**Objective:** To analyze gender differences on clinical effects of safinamide in PD patients treated in real-life conditions during the SYNAPSES trial.

**Methods:** SYNAPSES was a multinational, multicenter, observational study. At baseline, patients with PD diagnosis received safinamide as an add-on to levodopa and were followed up for 12 months, with visits performed every 4 months. A new statistical analysis was performed to describe the efficacy of safinamide in men and women on motor complications, motor symptoms, and adverse events.

**Results:** Six hundred and sixteen (38%) out of 1,610 patients enrolled in the SYNAPSES study were women and 994 (62%) men. Safinamide improved motor symptoms and motor complications (fluctuations and dyskinesia) in both genders, with a good safety profile and without requiring any change in the concomitant dopaminergic therapy. Clinically significant improvements, according to the criteria developed by Shulman et al., were seen in 46% of male and female patients for the UPDRS motor score and 43.5% of men vs. 39.1% of women for the UPDRS total score.

**Conclusions:** Safinamide was effective in improving motor fluctuations and dyskinesia and proved to be safe in both male and female patients with PD. Further prospective studies, specifically addressing potential gender differences in response to PD therapies, are needed to develop tailored management strategies.

## Introduction

Parkinson's disease (PD) is the second most common neurodegenerative disorder, affecting about 3% of the population by the age of 65 and up to 5% of the people over 85 years. The prevalence of PD is expected to rise dramatically over the next decades, with an increase in healthcare-related costs (1).

Typical PD motor manifestations include resting tremor, bradykinesia, rigidity, and gait impairment, but there are also several non-motor symptoms such as depression, anxiety, pain, orthostatic hypotension, sleep disorders, and gastrointestinal disturbances, which can precede the motor features by many years (2).

Together with aging, genetics, and environment, biological sex has been recognized as an important factor in the development of PD. Epidemiological studies showed that both incidence and prevalence of PD are 1.5–2 times higher in men than in women (3). Moreover, there are differences in the clinical presentation of both the motor and non-motor features of the disease. Women experience a later onset of motor symptoms and show more frequently a tremor-dominant phenotype associated with a slower disease progression and lower dopaminergic denervation (4), probably due to the neuroprotective effects of estrogens. Stiffness and behavioral and sleep disorders are more common in men (5). Male gender is a risk factor for the development of cognitive impairment and dementia in PD (6). Women more frequently present mood-related non-motor fluctuations (depression, anxiety) and pain, whereas impulse control disorders, such as pathological gambling and hypersexuality, are more common in male patients with PD (7, 8). Levodopa-related motor complications, such as dyskinesia and wearing-off, are more frequent in women, partly due to pharmacokinetic differences beyond a different weight (9). Considering these gender differences may help to tailor treatment, predict outcomes, and meet social needs in men and women with PD (8). In Italy, a law-regulating clinical trial with specific regard to the methodological approach of gender medicine was approved in 2018 (10).

**Figure 1:**
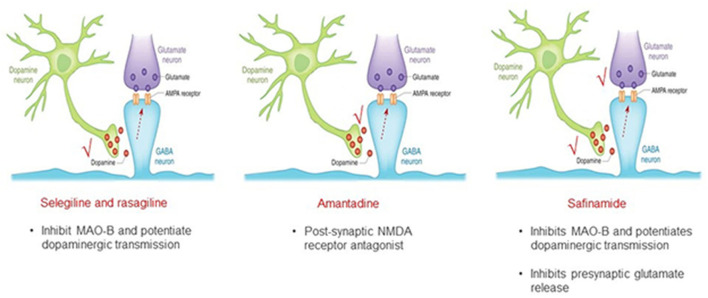
Safinamide is a multi-modal drug with a double mechanism of action.

Although the need for personalized medicine according to gender is now generally recognized, to date no gender-oriented advice is available for PD. Current therapies of PD are focused on symptomatic management and dopamine restoration. However, other neurotransmitters, including glutamate, are involved in the neurodegenerative process, clinical symptoms, and fluctuations of PD (11). Targeting a non-dopaminergic system may be an alternative strategy to improve the efficacy of levodopa and avoid its dose increase, with consequent potential worsening of motor and non-motor complications (12). Safinamide has a unique multimodal mechanism of action (MoA), dopaminergic and non-dopaminergic, that includes the reversible inhibition of the monoamine oxidase-B (MAO-B) enzyme and the modulation of excessive glutamate release (13). Safinamide differs from other pure MAO-B inhibitors like selegeline and rasagiline, and also from amantadine (Figure 1). This dual MoA might be important in terms of effect not only on motor symptoms, but also on motor complications, such as wearing-off and dyskinesia, and non-motor symptoms like pain and mood deterioration (14, 15).

Despite being the most frequently studied movement disorder, studies investigating the impact of gender on medical treatments in PD are still scarce. The aim of this study is to investigate the effects of safinamide according to gender in PD patients treated in real-life conditions during the SYNAPSES trial.

## Materials and Methods

### Study Design

This report describes the results of new additional analyses of the SYNAPSES trial (EU PAS Register Number EUPAS13745), a European multicentre, retrospective–prospective cohort observational study. The study was designed, in agreement with the European Agency (EMA), to investigate how safinamide is prescribed and used in routine clinical practice and to collect safety and efficacy data. The countries involved were Belgium, Germany, Italy, Spain, Switzerland, and United Kingdom, and the study was conducted in 128 Neurology and Geriatric centers specialized in PD treatment. A total of 1,610 patients with PD already receiving levodopa were enrolled. Patients were treated with safinamide as add-on therapy, according to the summary of product characteristics (SmPC), and followed up for 12 months after the start of treatment, with visits every 4 months. Male and female patients with PD were eligible, provided they were aged ≥18 years and started treatment with safinamide according to routine clinical practice. Patients were excluded if they were participating in any other clinical trial at study inclusion or in case of contraindications to safinamide as listed in the SmPC. According to the non-interventional study type, the Investigators were not given any guidelines regarding patient selection and treatment administration (16).

Both protocol and patient materials were approved by Independent Ethics Committees and Health Authorities of the participating countries. All patients signed informed and privacy consent forms and the study was conducted according to the ethical standards of the institutional and/or national research committee and according to the Declaration of Helsinki. Personal data were collected, stored, and processed exclusively in pseudoanymized form and in compliance with the regulatory requirements for the protection of confidentiality of patients.

### Data Source and Measurements

All patients' data were recorded by the Investigators in electronic standardized case report forms (eCRFs). Data on demography, adverse events (AEs), and motor complications were collected from the medical charts and by interviewing the patients at each study visit.

Age at onset and PD symptoms were retrieved from the patient's history, taken during the baseline visit. Age at onset was determined as the age at which the patient had first noticed any parkinsonian motor symptoms. Adverse event terms were coded with the Medical Dictionary for Regulatory Activities (MedDRA) version 21.1 (17). Seriousness, severity, and relation with safinamide were entered according to the clinicians' judgment.

Motor complications were recorded using a menu already available in the eCRF; based on the data derived from the SYNAPSES study, the Investigators, in agreement with the protocol and the statistical analysis plan approved by the European Agency (EMA), considered the following motor complications: any fluctuations, wearing-off, early morning fluctuations, unpredictable fluctuations, delayed on, and dyskinesia (16).

As requested by EMA, given the observational nature of the trial, the study was not designed to collect patients' diaries for ON and OFF periods or perform specific scales for motor complications that are not common in routine clinical practice.

Activities of daily living (ADL), motor symptoms, and complications of therapy were evaluated during ON time with the Unified Parkinson's Disease Rating Scale (UPDRS) (18), measuring the changes from baseline to each follow-up visit. The UPDRS part I score is not shown because, as described by Abbruzzese et al. (16), it was stable during the whole study. The UPDRS is a scale widely used by routine to follow the longitudinal clinical course of PD. The UPDRS total scores and the UPDRS part III (motor) scores were also evaluated following the criteria developed by Shulman et al. (19) for clinical significance. Moreover, the effects of safinamide on the cardinal motor symptoms were investigated separately with this questionnaire, specifically bradykinesia (UPDRS items 23,24,25,26,31), rigidity (UPDRS item 22), tremor (UPDRS items 16,20,21), and postural instability and gait disorder (PIGD, UPDRS items 13,14,15,29,30) (20).

### Statistical Methods

The statistical analyses were done on all “evaluable patients for the full analysis set” defined as the patients satisfying all inclusion criteria and not violating any exclusion criteria. Categorical variables were described by means of absolute and relative frequencies, while continuous variables by means of mean, standard deviation, quartiles, minimum, and maximum. Response variables recorded at baseline and each follow-up visit were analyzed using generalized estimating equation (GEE) models with identity link function and Gaussian distribution for UPDRS total score, UPDRS subscales scores, and cardinal motor symptoms scores, and also binomial distribution for the rate of motor complications (fluctuations and dyskinesia). Baseline, gender, visit, and gender-by-visit interaction were applied as model fixed effects. The variance-covariance matrix of the GEE model, which takes into account correlation across repeated measures, was parameterized using the first-order autoregressive form. The estimates of changes from baseline at each follow-up visit were computed using proper contrasts applied on the gender-by-visit interaction and were used to test the differences between the genders. Quasi-maximum likelihood estimates of the model parameters were obtained with the GEE procedure of SAS software Version 9.4. Results are reported as model-based estimates [least squares (LS) mean scores or LS proportions depending on the response variable] with standard errors. Conventional Chi-square test or Fisher's exact test, if deemed more appropriate, were used to test gender differences regarding levodopa assumption (increase/decrease) and the incidence of AEs.

## Results

### Demography

The patients' overview by gender is shown in Table 1. Out of the 1,610 patients enrolled in the SYNAPSES study, 616 (38%) were women and 994 (62%) men. There were no differences as for “time to PD diagnosis” (about 8 years), “disease duration” (about 9 years), “race” (99% were Caucasian), and “Hoehn and Yahr (H&Y) stage” (21) (about 95% of patients had H&Y stage ≥ 2). Women were older than men at enrollment (69.4 years women vs. 67.8 years men, *p* = 0.0007) and the onset of symptoms (60.2 years women vs. 58.6 men, *p* = 0.0088).

**Table 1:** Patients' overview by gender.

		**All population (*N* = 1,610)**	**Females (*N* = 616)**	**Males (*N* = 994)**	***P*-value**
Age at enrollment (years)	Mean (SD)	68.4 (9.7)	69.4 (9.4)	67.8 (9.7)	0.0007
Race (*N*, %)	Caucasian	1,593 (99.0%)	610 (99.0%)	983 (98.9%)	0.3845
	Other	17 (1.0%)	6 (1.0%)	11 (1.1%)	
Diagnosis (*N*, %)	Idiopathic PD	1,593 (99.0%)	612 (99.4%)	981 (98.7%)	0.4516
	Atypical Parkinsonisms	13 (0.8%)	3 (0.5%)	10 (1.0%)	
	Other^*^	4 (0.2%)	1 (0.1%)	3 (0.3%)	
Time from PD diagnosis (years): mean (SD)		7.9 (5.4)	8.0 (5.5)	7.9 (5.3)	0.5887
Disease duration (years): mean (SD)		8.8 (5.6)	8.9 (5.5)	8.8 (5.6)	0.5937
Age at onset of symptoms (years): mean (SD)		59.2 (11.0)	60.2 (11.0)	58.6 (11.0)	0.0088
Hoehn and Yahr stage	1	86 (5.3%)	32 (5.2%)	54 (5.4%)	0.1215
	2	818 (50.8%)	293 (47.6%)	525 (52.8%)	
	3	437 (27.1%)	185 (30.0%)	252 (25.4%)	
	4	88 (5.5%)	41 (6.7%)	47 (4.7%)	
	5	6 (0,4%)	3 (0.5%)	3 (0.3%)	
	Missing	175 (10.9%)	62 (10.0%)	113 (11.4%)	

*Percentages (%) were computed by column*.

*N, number of patients; SD, Standard Deviation; PD, Parkinson's Disease*.

* *Patients with other diagnoses had juvenile Parkinsons' disease*.

### UPDRS Scores

**Figure 2:**
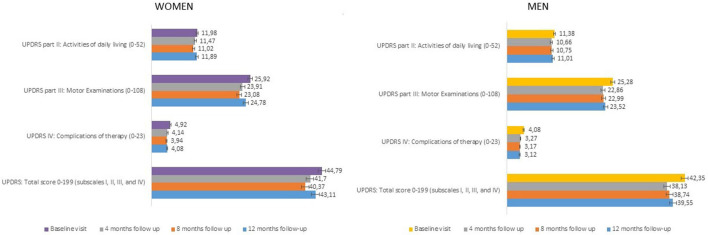
UPDRS scores (part II, III, IV, total scores) at each study visit (± SE). UPDRS, Unified Parkinson's Disease Rating Scale; SE, Standard Error.

Changes from baseline at each follow-up visit in UPDRS total score and UPDRS subscales scores (part II ADL, part III Motor Examination, part IV Complications of Therapy) are presented in Figure 2 (women and men). At baseline, women presented higher UPDRS scores compared to men, particularly for the total score (44.79 vs. 42.35, respectively) and the part IV, complications of therapy (4.92 vs. 4.08, respectively). Nevertheless, these differences were not statistically significant (Table 2). During the study, improvements were seen in all follow-up visits for both genders, starting already from the 4-month visit. The UPDRS total score, the UPDRS part II (ADL), and the UPDRS part III (motor examination) improved up to 10%, while the highest improvement (up to 20%) was detected in UPDRS part IV (complications of therapy), but did not show significant differences between men and women (Table 2).

**Table 2:** UPDRS changes from baseline at each follow-up visit in female vs. male patients.

**UPDRS subscale**	**Follow-up visit**	**Female vs. male difference in change from baseline^†^**	**Standard error**	***P*-value**
II—ADL	4 months	0.2163	0.5807	0.7096
II—ADL	8 months	−0.3301	0.6022	0.5836
II—ADL	12 months	0.2848	0.6127	0.6421
III—Motor examination	4 months	0.4068	1.0959	0.7105
III—Motor examination	8 months	−0.5471	1.112	0.6227
III—Motor examination	12 months	0.6257	1.1334	0.5809
IV -Complications of therapy	4 months	0.02664	0.2536	0.9163
IV -Complications of therapy	8 months	−0.0714	0.2607	0.7842
IV -Complications of therapy	12 months	0.1142	0.2631	0.6644
Total score (I, II, III, and IV)	4 months	1.1295	1.7901	0.5281
Total score (I, II, III, and IV)	8 months	−0.8101	1.8325	0.6585
Total score (I, II, III, and IV)	12 months	1.1214	1.8793	0.5507

† *Difference between mean female score and mean male score at follow-up visits adjusted for the difference between mean female score and mean male score at baseline*.

*UPDRS, Unified Parkinson's disease rating scale; ADL, Activities of daily living*.

**Figure 3:**
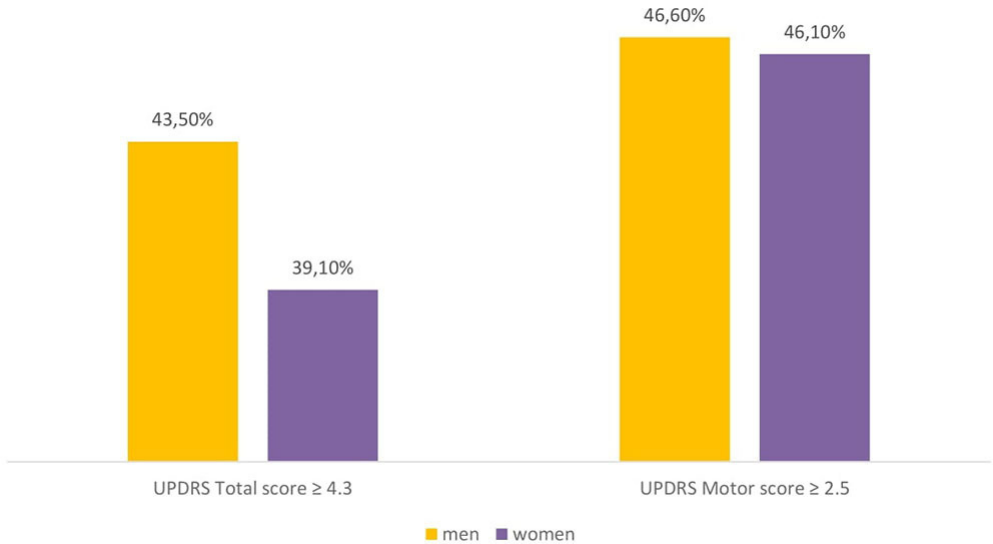
Percentage of male and female patients with clinically important difference (improvement) in the UPDRS scores (difference between 12-months follow-up and baseline). UPDRS, Unified Parkinson's Disease Rating Scale.

The percentage of patients with clinically relevant differences, according to Shulman et al. (19), in the UPDRS part III score (>2.5 points) and UPDRS total score (> 4.3 points) is reported in Figure 3. At the end of the study, the same percentage of subjects showed a clinically significant improvement in UPDRS part III scores (men: 46.6%; women: 46.1%, *p*: 0.90266), while the clinical improvement on the UPDRS total score was slightly higher in men than women (43.5 vs. 39.1%, respectively), even if there was no statistical difference between genders (*p*: 0.29831).

### Cardinal Motor Symptoms

At baseline, bradykinesia and PIGD were more severe in women, while rigidity was more severe in men. These differences were not statistically significant. Tremor showed the same severity in both genders. All cardinal symptoms improved since the first follow-up visit. Tremor showed the highest improvement (up to 23% in men and up to 24% in women). Bradykinesia improved up to 11% and rigidity up to 10% in both genders. PIGD improved up to 8% in men and up to 11% in women (Table 3). As shown in Table 4, no gender differences were detected.

**Table 3:** Descriptive statistics of cardinal motor symptoms grouped for gender and visit.

**Motor symptom**	**Gender**	**Visit**	**Estimate**	**Standard error**	**Lower 95% CL**	**Upper 95% CL**
Bradykinesia	Female	Baseline	11.89	0.30	11.31	12.47
Bradykinesia	Female	4 Months	11.13	0.28	10.59	11.68
Bradykinesia	Female	8 Months	10.56	0.30	9.98	11.14
Bradykinesia	Female	12 Months	11.32	0.30	10.73	11.91
Bradykinesia	Male	Baseline	11.01	0.23	10.56	11.45
Bradykinesia	Male	4 Months	9.84	0.22	9.41	10.26
Bradykinesia	Male	8 Months	10.21	0.23	9.76	10.66
Bradykinesia	Male	12 Months	10.27	0.23	9.82	10.73
PIGD	Female	Baseline	5.17	0.18	4.82	5.52
PIGD	Female	4 Months	4.90	0.16	4.58	5.22
PIGD	Female	8 Months	4.62	0.18	4.27	4.96
PIGD	Female	12 Months	4.87	0.18	4.51	5.22
PIGD	Male	Baseline	4.17	0.14	3.90	4.44
PIGD	Male	4 Months	3.85	0.13	3.60	4.10
PIGD	Male	8 Months	3.95	0.14	3.68	4.22
PIGD	Male	12 Months	4.13	0.14	3.85	4.41
Rigidity	Female	Baseline	5.04	0.17	4.71	5.38
Rigidity	Female	4 Months	4.62	0.17	4.29	4.94
Rigidity	Female	8 Months	4.54	0.17	4.20	4.88
Rigidity	Female	12 Months	4.78	0.17	4.44	5.12
Rigidity	Male	Baseline	5.52	0.13	5.25	5.78
Rigidity	Male	4 Months	4.97	0.13	4.72	5.23
Rigidity	Male	8 Months	5.00	0.13	4.74	5.26
Rigidity	Male	12 Months	5.15	0.13	4.89	5.41
Tremor	Female	Baseline	3.36	0.19	3.00	3.73
Tremor	Female	4 Months	2.55	0.16	2.23	2.86
Tremor	Female	8 Months	2.45	0.16	2.14	2.77
Tremor	Female	12 Months	2.60	0.17	2.26	2.94
Tremor	Male	Baseline	3.34	0.15	3.05	3.63
Tremor	Male	4 Months	2.80	0.13	2.55	3.04
Tremor	Male	8 Months	2.57	0.12	2.33	2.82
Tremor	Male	12 Months	2.57	0.13	2.31	2.83

*PIGD, Postural instability gait disorder*.

**Table 4:** Changes of cardinal motor symptoms scores from baseline at each follow-up visit in female vs. male patients.

**Motor symptom**	**Follow-up visit**	**Female vs. male difference in change from baseline^†^**	**Standard error**	***P*-value**
Bradykinesia	4 months	0.4125	0.5138	0.4221
Bradykinesia	8 months	−0.5340	0.5290	0.3128
Bradykinesia	12 months	0.1671	0.5334	0.7541
PIGD	4 months	0.0461	0.3066	0.8806
PIGD	8 months	−0.3300	0.3166	0.2973
PIGD	12 months	−0.2621	0.3221	0.4159
Rigidity	4 months	0.1183	0.3041	0.6974
Rigidity	8 months	0.0152	0.3088	0.9609
Rigidity	12 months	0.1012	0.3105	0.7446
Tremor	4 months	−0.2734	0.3148	0.3851
Tremor	8 months	−0.1440	0.3142	0.6469
Tremor	12 months	0.0092	0.3240	0.9775

† *Difference between mean female score and mean male score at follow-up visits adjusted for the difference between a mean female score and mean male score at baseline*.

*PIGD, Postural instability gait disorder*.

### Levodopa Dose

During the study, the overall mean dose of levodopa (alone or associated with catechol-*O*-methyltransferase inhibitors) did not change significantly. The mean levodopa (L-dopa) daily dose at baseline was 425 mg (300–550 mg) and the mean daily dosage at the end of the study was 450 mg (300–600 mg). There were no differences between the genders both for baseline and follow-up L-dopa daily doses.

### Motor Complications (Fluctuations and Dyskinesia)

**Figure 4:**
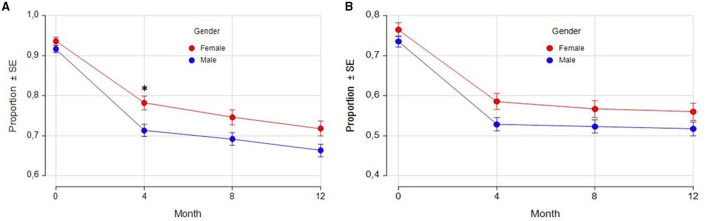
Any fluctuations **(A)** and wearing-off **(B)**: proportion of patients by gender and visits. SE, Standard Error. *p = 0.0210 (difference between female and male proportions at follow-up visits adjusted for the difference between female and male proportions at baseline).

Fluctuations have been recorded in the eCRF by the Investigators as “any fluctuations,” “wearing off,” “early morning fluctuations,” “unpredictable fluctuations,” and “delayed ON.” As shown in Table 5, at baseline the majority of patients had motor fluctuations, with a slightly higher percentage in women (93.6%) compared with men (91.6%). The most frequent one was “wearing off,” again with a mild prevalence in women (76.5 vs. 73.5% in men). Also “early morning fluctuations” were more frequent in women (25.7 vs. 21.5% in men), while there was the same prevalence between the genders for “unpredictable fluctuations” and “delayed ON.” The prevalences of different fluctuations at baseline were not significantly different between genders. The percentage of patients with motor fluctuations decreased in both genders during the study since the 4-month follow-up visit, thus indicating a rapid effect of safinamide. At 12 months, the percentage decreased to 24% (women) and 28% (men) for “any fluctuations”; 27% (women) and 30% (men) for “wearing off”; 44% (women) and 40% (men) for “early morning fluctuations”; 37% (women) and 43% (men) for “unpredictable fluctuations”; and 32% (women) and 22% (men) for “delayed ON.” In Figure 4 the proportion of patients at each study visit for “any fluctuations” (A) and “wearing off” (B) are shown graphically. There were no statistically significant differences in changes from baseline except for “any fluctuations” at 4-month follow-up, where there was a higher percentage reduction of prevalence in men as compared with women (22 vs. 16%, respectively, *p* = 0.0210).

**Table 5:** Prevalence of fluctuations and dyskinesia according to gender at the start of treatment with safinamide and during the follow-up.

**Motor complications**	**Gender**	**Baseline**	**4 months**	**8 months**	**12 months**
Any fluctuations	Female	93.6%	78.2%	74.6%	71.8%
	Male	91.6%	71.3%	69.2%	66.3%
Wearing-off	Female	76.5%	58.5%	56.7%	56.0%
	Male	73.5%	52.8%	52.3%	51.7%
Dyskinesia	Female	48.8%	44.6%	39.9%	35.1%
	Male	33.2%	28.3%	26.6%	23.3%
Early morning fluctuations	Female	25.7%	15.1%	16.8%	14.4%
	Male	21.5%	13.0%	13.5%	13.1%
Unpredictable fluctuations	Female	17.5%	11.0%	11.1%	11.0%
	Male	16.2%	11.2%	11.2%	9.2%
Delayed ON	Female	11.9%	9.8%	8.5%	8.1%
	Male	11.0%	7.2%	7.8%	8.6%

*Percentages (%) were computed by column*.

**Figure 5:**
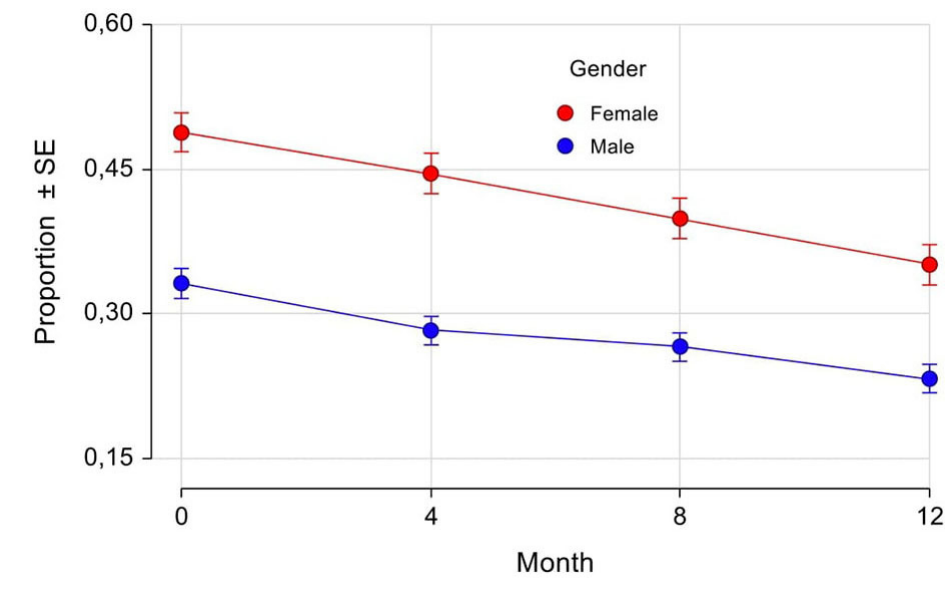
Dyskinesia: Proportion of patients by gender and visits. SE, Standard Error.

At baseline, dyskinesia had the highest prevalence in women compared with males (48.8 vs. 33.2%, respectively, Table 5), even if not statistically significant. Safinamide reduced the percentage of patients with dyskinesia in both genders up to 30%, with noticeable effects already at 4 months follow-up (refer to Table 5, Figure 5). No gender differences were detected at any study visit.

### Adverse Events and Serious AEs

As reported in Table 6, during observation 295 (47.9%) female patients and 437 (44.0%) male patients experienced at least one AE. This difference was not statistically significant. The majority of AEs were rated as mild or moderate and were those already described in the patients' leaflet (16). The most frequent AE was dyskinesia, with a higher prevalence in women (14.3%) compared with men (8.7%; *p* < 0.05). Dyskinesia was generally transient and did not lead to drug discontinuations. Other AEs with a frequency ≤ 3% of the total number of events were hallucinations (3.1% in women vs. 2.7% in men), dizziness (2.3% in women vs. 2.4% in men), sleep disorders (1.9% in women vs. 2.3% in men), mood disorders (2.8% in women vs. 1.3% in men), and nausea (2.1% in women vs. 0.5% in men; *p* < 0.05). There were no statically significant differences between genders except for dyskinesia and nausea, but none of the above AEs was considered related to safinamide treatment by the clinicians.

**Table 6:** Summary of treatment emergent adverse events (TEAEs) according to gender.

	**Females (*n* = 616)**	**Males (*n* = 994)**	***P*-value^†^**
Any TEAEs	295 (47.9%)	437 (44.0%)	n.s.
Dyskinesia	88 (14.3%)	86 (8.7%)	<0.05
Dizziness	19 (3.1%)	27 (2.7%)	n.s.
Hallucinations	14 (2.3%)	24 (2.4%)	n.s.
Sleep disorders	12 (1.9%)	23 (2.3%)	n.s.
Mood disorders	17 (2.8%)	13 (1.3%)	n.s.
Nausea	13 (2.1%)	5 (0.5%)	<0.05

*Each subject is counted at most once within each primary system organ class and preferred term*.

*Percentages are calculated on the number of subjects (n) in the safety analysis set by investigational product*.

*n, number of subjects; %, percentages of subjects; n.s., not significant*.

† *Two-sided p-value*.

Serious adverse events (SAEs) occurred in 9.6% of women and 8.9% of men. This difference was not statistically significant. The reason for reporting SAEs in these patients was a new or prolonged disability. The most frequently reported SAE was dyskinesia, with the same percentage in both genders (0.2%). As already reported by Abbruzzese et al. (16), the majority of SAEs were completely resolved at follow-up, and no SAEs were considered related to safinamide treatment.

## Discussion

Increased recognition of PD sex-based differences could help to develop tailored approaches for patients' care (22). These additional analyses of the SYNAPSES study enable for the first time the assessment of the effects of safinamide across genders.

The observed 2-year difference in age at PD onset and enrollment between men and women is consistent with most epidemiological studies, suggesting that the development of symptomatic PD is slightly delayed in women compared with men (3). This could be explained by higher initial striatal dopamine levels in women that could delay the dopamine depletion and hence postpone the development of parkinsonian symptoms. Moreover, estrogens are supposed to play an important role in differences between male and female patients (4, 23).

At baseline, women showed a more severe postural instability and bradykinesia compared to men; rigidity was more severe in men, whereas the severity of tremor was similar. It is acknowledged that bradykinesia and rigidity are most responsive to levodopa, which has a limited effect on postural stability, gait, and tremor (24). The addition of safinamide improved all cardinal symptoms in both genders. Consistent with these benefits, UPDRS scores improved with safinamide with a clinically important difference (CID) in the motor scores (19) in 46% of men and women. A CID is the change that patients recognize as clinically valuable and is one of the most important tools for patient-centered trials (25). A similar improvement was seen in the activities of daily living, measured by the UPDRS II. These benefits are particularly important for women because they are less likely to have informal caregiver support (26). Overall, these results are noteworthy because patients were already receiving a concomitant dopaminergic therapy, and the improvement was not related to changes in the levodopa dose that was found to be stable during the study.

The occurrence of dyskinesia and motor fluctuations, such as wearing-off (WO), is a fundamental problem in the long-term management of patients with PD. A higher prevalence of levodopa-induced dyskinesia and WO has been reported in women vs. men with PD (6, 26) and is confirmed also in our study. WO is associated with a poorer quality of life and frequently extends beyond motor impairment to non-motor domains (27). Safinamide, as adjunct therapy, significantly reduced motor fluctuations, particularly WO, and dyskinesia in both genders, with a rapid-onset effect that may be explained by glutamate modulation. Glutamate and other neurotransmitters, in addition to dopamine, are known to contribute to the appearance of motor complications (28). The statistical difference between genders at month 4 for “any fluctuations” was not confirmed in the later visits and the clinical significance is not clear. Evidence reports a shorter time to develop WO in women compared with men (29, 30). We could speculate that a longer presence of motor fluctuations in women can account for this difference in time to get a similar benefit from safinamide on the reduction of motor fluctuations. Unfortunately, we have no data about the time from PD onset to the occurrence of motor fluctuations in our population.

The positive effects on both motor fluctuations and dyskinesia are in line with previous reports showing that safinamide has stronger efficacy than rasagiline on fluctuations and dyskinesia (16, 31), and are reflected also by the improvement found in both genders in the UPDRS part IV assessing the complications of the therapy.

Safinamide was safe and well-tolerated in both genders. As reported in the literature, women had a higher prevalence of dyskinesia and nausea compared with men, although at a lower frequency than those observed in the interventional clinical trials (9, 32). No gender differences were detected as for serious adverse events nor the causal relationship with safinamide.

We must acknowledge some limitations of our study, such as the retrospective design of the trial and the lack of standardized scales or patients' diaries assessing motor complications.

Moreover, stratifications according to the administration of PD medications other than safinamide in addition to levodopa were not feasible since all patients had ≥ 2 concomitant baseline treatments; thus treatment subgroups partly overlapped. Finally, the study was not powered to discriminate between different doses of safinamide, and for this reason, the data of the patients were pooled irrespective of the dose administered. However, these limitations were counterbalanced by the large sample size of fluctuating patients in all stages of the disease, the real-life evaluation, and the high number of outpatient clinics involved in different European countries.

## Conclusions

No studies are yet available about gender differences in the response to anticholinergics, catechol-*O*-methyltransferase inhibitors, and MAO-B inhibitors, nor recommendations have been formulated about a gender-tailored medical treatment in PD.

Our additional analysis of the SYNAPSES study has shown no significant gender differences on the efficacy of safinamide in fluctuating PD patients, suggesting that safinamide might improve motor complications in both genders with no changes in the concomitant dopaminergic therapy. Moreover, this study confirms the good tolerability of safinamide in both genders, making safinamide a safe and effective option to treat motor complications of PD in both men and women.

Future prospective studies, specifically addressing gender differences in response to antiparkinsonian drugs, are needed to develop tailored-management strategies in PD.

## Data Availability Statement

The raw data supporting the conclusions of this article will be made available by the authors, without undue reservation.

## Ethics Statement

The study was designed, in agreement with the European Agency (EMA), to investigate how safinamide is prescribed and used in routine clinical practice and efficacy data. The countries involved were Belgium, Germany, Italy, Spain, Switzerland, and United Kingdom and the study was conducted in 128 Neurology and Geriatric centers specialized in PD treatment (the list available as a supplementary file). Both protocol and patient materials were approved by Independent Ethics Committees and Health Authorities of the participating countries. All patients signed informed and privacy consent forms and the study was conducted according to the ethical standards of the institutional and/or national research committee and according to the Declaration of Helsinki. Personal data were collected, stored, and processed exclusively in pseudonymized form and in compliance with the regulatory requirements for the protection of confidentiality of patients. The patients/participants provided their written informed consent to participate in this study.

## Author Contributions

All authors contributed to writing and reviewing the manuscript and approved the final manuscript.

## Conflict of Interest

MTP, MP, MR, and MD are members of the Scientific Advisory Board of Zambon S.p.A. CC and IM are employees at Zambon S.p.A. EB is a statistical consultant for Zambon S.p.A. Zambon S.p.A. was involved in the study design and collection of data for the original study. Zambon S.p.A funded the original study. CC requested approval for publication from Zambon S.p.A as an employee but Zambon S.p.A was not involved in the analysis, interpretation of data, writing of this article, or the decision to submit it for publication.

## Publisher's Note

All claims expressed in this article are solely those of the authors and do not necessarily represent those of their affiliated organizations, or those of the publisher, the editors and the reviewers. Any product that may be evaluated in this article, or claim that may be made by its manufacturer, is not guaranteed or endorsed by the publisher.
