# Supplementary material for: Efficacy of Safinamide and Gender Differences During Routine Clinical Practice
Source: Front Neurol. 2021 Dec 14;12:756304. doi: 10.3389/fneur.2021.756304 (PMC8712933; doi:10.3389/fneur.2021.756304)
Supplement: Supplementary file 1 [file Table_1.docx]

| **Country** | **Institution** | **City** |
| --- | --- | --- |
| Italy | P.O. San Salvatore - Centro Sclerosi Multipla Clinica Neurologica | L'Aquila |
| Italy | IDC Capodimonte – Neurologia | Napoli |
| Italy | A.O.U. Seconda Università di Napoli - I Clinica Neurologica | Napoli |
| Italy | A.O.U. OO.RR. S.Giovanni di Dio e Ruggi D'Aragona – Neurologia | Salerno |
| Italy | Azienda Ospedaliera Universitaria - Neurologia | Parma |
| Italy | Arcispedale Sant'Anna – Neurologia | Cona |
| Italy | Ospedale Bellaria – Neurologia | Bologna |
| Italy | Nuovo Ospedale Sant'Agostino-Estense - Clinica Neurologica | Modena |
| Italy | A.O.U. S.Maria della Misericordia - SOC Neurologia | Udine |
| Italy | Ospedale San Raffaele Cassino - U.O. Riabilitazione Neuromotoria | Cassino |
| Italy | Università Cattolica S. Cuore Policlinico Gemelli – Neurologia | Roma |
| Italy | Università Campus Bio-Medico – Neurologia | Roma |
| Italy | A.O. Sant' Andrea - Univ. La Sapienza - U.O.C. Neurologia | Roma |
| Italy | Policlinico Umberto I - Univ La Sapienza - Neurologia B - Dip. Scienze Neurologiche | Roma |
| Italy | Ospedale San Giovanni Battista – Neurologia | Roma |
| Italy | Policlinico Tor Vergata - Clinica Neurologica | Roma |
| Italy | Università degli Studi di Genova - Centro Parkinson - Dipartimento DINOGMI | Genova |
| Italy | ASST Gaetano Pini-CTO - Centro per la malattia di Parkinson e disturbi del movimento | Milano |
| Italy | Ospedale San Raffaele - Dipartimento Neurologico | Milano |
| Italy | ASST Spedali Civili - U.O. 2^ Neurologia | Brescia |
| Italy | ASST Santi Paolo e Carlo – Neurologia | Milano |
| Italy | Istituto Neurologico C. Mondino - U.O. Parkinson | Pavia |
| Italy | A.O.U. Ospedali Riuniti - Clinica Neurologia | Ancona |
| Italy | Fondazione Univ. D'Annunzio - Centro Ricerche Cliniche | Chieti |
| Italy | Istituto Neurologico Mediterraneo Neuromed – Neurologia | Pozzilli |
| Italy | A.S.O. Molinette - Neurologia 2 | Torino |
| Italy | Ospedale Cardinal Massaia – Neurologia | Asti |
| Italy | Ospedale Generale Regionale F.Miulli - U.O.C. Neurologia | Acquaviva delle Fonti |
| Italy | A.O.U. Policlinico Consorziale - U.O. Neurofisiopatologia | Bari |
| Italy | Azienda Ospedaliera G. Brotzu - U.O. Neurologia | Cagliari |
| Italy | A.O.U. Policlinico Monserrato – Neurologia | Monserrato |
| Italy | A.O Riuniti Villa Sofia-Cervello - Neurologia Centro Rif.Regionale Parkinson | Palermo |
| Italy | A.O.U. Policlinico-Vittorio Emanuele - Clinica Neurologica | Catania |
| Italy | A.O.U. Policlinico G. Martino - UOSD Neurofisiopatologia e Disordini del Movimento | Messina |
| Italy | Ospedale della Misericordia – Neurologia | Grosseto |
| Italy | A.O.U. Pisana Ospedale Santa Chiara - U.O. Neurologia | Pisa |
| Italy | Ospedale S. Maria della Misericordia - Clinica Neurologica | Perugia |
| Italy | Ospedale San Camillo - U.O. Malattia di Parkinson | Venezia Lido |
| Italy | Ospedale dell'Angelo – Neurologia | Mestre |
| Italy | Casa di Cura Villa Margherita – Neurologia | Arcugnano |
| Italy | Policlinico - Borgo Roma - U.O. Neurologia B | Verona |
| Italy | Ospedale San Salvatore – Neurologia | Pesaro |
| Italy | A.O.U. Policlinico P. Giaccone – Neurologia | Palermo |
| Italy | Casa di Cura San Francesco – Neurologia | Bergamo |
| Italy | Ospedale civile S.Maria delle Croci – Neurologia | Ravenna |
| Italy | Ospedale S. Giovanni di Dio – Neurologia | Crotone |
| Italy | Azienda Ospedaliera Universitaria Integrata – Neurologia | Trieste |
| Italy | A.O.U. Maggiore della Carità – Neurologia | Novara |
| Italy | Ospedale di Circolo e Fondazione Macchi – Neurologia | Varese |
| Italy | Ospedale San Martino – Neurologia | Belluno |
| Italy | A.O. Santa Maria - U.O.C. Neurologia | Terni |
| Italy | Istituto Chirurgico Ortopedico Traumatologico - Riabilitazione Neurologica | Latina |
| Italy | Ospedale A. Perrino – U.O.C. Neurologia | Brindisi |
| Spain | Hospital Puerta de Hierro | Majadahonda |
| Spain | Hospital de Cruces | Barakaldo |
| Spain | Hospital de Donostia | Donostia |
| Spain | Hospital Ramón y Cajal | Madrid |
| Spain | Hospital Insular de Las Palmas | Las Palmas |
| Spain | Hospital Arquitecto Marcide | El Ferrol |
| Spain | Hospital Universitario Virgen Macarena | Sevilla |
| Spain | Hospital Universitari de Bellvitge | L'Hospitalet de Llobregat |
| Spain | Hospital Univeritario Puerta del Mar | Cadiz |
| Spain | Hospital Univeritario La Princesa | Madrid |
| Spain | Xanit Hospital Internacional | Benalmádena |
| Spain | Hospital General Universitario de Ciudad Real | Ciudad Real |
| Spain | Hospital Universitari Vall d'Hebron | Barcelona |
| Spain | Hospital Quirón-Teknon | Barcelona |
| Spain | Hospital Universitari Son Espases | Palma de Mallorca |
| Spain | Fundación Investigación Hospital General Universitario de Valencia | Valencia |
| Spain | Hospital Universitari i Politècnic La Fe | Valencia |
| Spain | Hospital General Universitario de Alicante | Alicante |
| Spain | Hospital Público Universitario Del Henares | Coslada (Madrid) |
| Spain | Hospital de Tortosa Verge de la Cinta | Tortosa |
| Spain | Hospital General Universitario de Elche | Elche |
| Spain | Hospital de la Santa Creu i Sant Pau | Barcelona |
| Spain | Ruber Internacional | Madrid |
| Spain | Hospital Universitario de Burgos | Burgos |
| Spain | Hospital Virgen de la Salud | Toledo |
| Spain | Clínic Universitari Barcelona | Barcelona |
| Spain | Hospital Lozano Blesa | Zaragoza |
| Spain | Clínica Universidad de Navarra | Pamplona |
| Spain | Hospital Dexeus | Barcelona |
| Spain | Hospital Gregorio Marañon | Madrid |
| Spain | Hospital Virgen del Rocío | Sevilla |
| Spain | Hospital Universitario Infanta Sofía | Madrid |
| Spain | Hospital Universitario Infanta Leonor | Madrid |
| Spain | Hospital Universitario Locus Augusti de Lugo | Lugo |
| Spain | Hospital Universitario Central de Asturias | Oviedo |
| Spain | Centro de Neurología Avanzada | Sevilla |
| Spain | Hospital Santa Caterina | Salt - Girona |
| Spain | Hospital Parc Sanitari Sant Joan | Sant Boi de Llobregat |
| Germany | Gemeinschaftspraxis für Neurologie, Psychiatrie und Psychotherapie Dres. Gerlach und Kollegen | Freiburg |
| Germany | Neurozentrum Stuttgart Mitte | Stuttgart |
| Germany | Katholisches Klinikum Bochum gGmbH Neurologische Klinik | Bochum |
| Germany | Neurologie Bewegt Ärztehaus BISMARCK KARREE | Berlin |
| Germany | Nervenärztliche Gemeinschaftspraxis NeuroPoint GmbH | Ulm |
| Germany | Gesundheitszentrum Hoppegarten Praxis für Neurologie | Hoppegarten |
| Germany | St. Josef-Krankenhaus Kupferdreh Neurologie | Essen |
| Germany | Kliniken Kreis Mühldorf am Inn, Parkinson und andere Bewegungsstörungen, Abteilung f. klinische Studien | Haag in Oberbayern |
| Germany | Praxis für Psychiatrie und Neurologie | Gera |
| Germany | Universitätsklinikum Münster, Klinik für Allgemeine Neurologie | Münster |
| Germany | Parkinson-Klinik Ortenau GmbH & Co. KG, Zentrum für neurologische Bewegungsstörungen | Wolfach |
| Germany | Praxis für Neurologie | Regensburg |
| Germany | Neurologische Praxis Siegen | Siegen |
| Germany | Neurologische Facharztpraxis | Bochum |
| Belgium | Universitair Ziekenhuis Antwerpen | Edegem (Antwerp) |
| Belgium | Centre Hospitalier Universitaire Sart Tilman | Liège |
| Belgium | Universitair Ziekenhuis Gent | Gent |
| Belgium | Hôpital Saint-Luc | Louvain (Brussels) |
| Belgium | Hôpital Erasme | Brussels |
| Belgium | AZ Delta campus Wilgenstraat | Roeselare |
| Belgium | CHU Tivoli | La Louvière |
| Belgium | AZ Sint-Jan | Brugge |
| Belgium | Sint Lucas Ziekenhuis | Gent |
| Belgium | Virga Jesse Ziekenhuis | Hasselt |
| Belgium | Algemeen Ziekenhuis Klina | Antwerp |
| Belgium | Clinique St. Pierre | Ottignies |
| Belgium | CHU UCL Namur | Dinant |
| UK | Royal United Hospitals Bath NHS Foundation Trust | Bath |
| UK | Greater Manchester Neuroscience Centre, Salford Royal NHS Foundation Trust | Salford |
| UK | Livewell Southwest / Royal Devon & Exeter NHS Foundation Trust | Plymouth |
| UK | Fairfield General Hospital, Ward 19 Clinical Trials Unit | Bury |
| UK | Princess of Wales Hospital | Wales |
| UK | Kings Mill hospital | Mansfield |
| Switzerland | Luzerner Kantonsspital Neurology and Neuroabilitation | Lucerne |
| Switzerland | Inselspital Universitätsklinik für Neurologie | Bern |
| Switzerland | Kantonsspital St. Gallen Neurology Clinic | St.Gallen |
| Switzerland | Ospedale Civico, Neurocentro della Svizzera Italiana | Lugano |
| Switzerland | Kantonsspital Fribourg Service de neurologie | Fribourg |
